# Hybrid Porous Silicon Biosensors Using Plasmonic and Fluorescent Nanomaterials: A Mini Review

**DOI:** 10.3389/fchem.2020.00454

**Published:** 2020-05-29

**Authors:** Nedal Abu-Thabit, Elaref Ratemi

**Affiliations:** Department of Chemical and Process Engineering Technology, Jubail Industrial City, Al Jubail, Saudi Arabia

**Keywords:** porous silicon, biosensor, plasmonic, quantum dots, carbon dots, fluorescence, dual-mode

## Abstract

During the last two decades, porous silicon (PSi) has been proposed as a high-performance biosensing platform due to its biocompatibility, surface tailorability, and reproducibility. This review focuses on the recent developments and progress in the area related to hybrid PSi biosensors using plasmonic metal nanoparticles (MNPs), fluorescent quantum dots (QDs), or a combination of both MNPs and QDs for creating hybrid nanostructured architectures for ultrasensitive detection of biomolecules. The review discusses the mechanisms of sensitivity enhancement based on Localized Surface Plasmon Resonance (LSPR) of MNPs, Fluorescence Resonance Energy Transfer (FRET) in the case of MNPs/QDs donor-acceptor interactions, and photoluminescence/fluorescence enhancement resulting from the embedded fluorescent QDs inside the PSi microcavity. The review highlights the key features of hybrid PSi/MNPs/QDs biosensors for dual-mode detection applications.

## Introduction

Porous silicon (PSi) was accidentally discovered in 1956 by Arthur Uhlir and Ingeborg Uhlir, a husband and wife team working at Bell Laboratories. During one of the electro-polishing experiments, fine holes were formed and propagated in the <100> direction of the silicon wafer (Sailor, 2012). This observation was reported as a technical note without further investigation. In 1960, Allen Gee reported a faint reddish glow from a stain-etched porous silicon sample, with no explanation for the mechanism of the observed light emission (Sailor, 2012). PSi gained remarkable attention as a novel nanostructured photonic material in the early 1990s after the Canham's discovery, which reported strong and bright red-orange photoluminescence from PSi at room temperature (Canham, 1990). The efficient multicolor emission from PSi is attributed to the presence of nanometer-scale quantum wires as predicted by the quantum confinement model, which confirmed that the optical physics of the nanomaterial differs from bulk crystalline silicon (Cullis and Canham, 1991). In 1997, the Sailor research group reported label-free detection of oligonucleotide target using PSi Fabry-Pérot interferometer (Lin et al., 1997). Since then, PSi nanostructures attracted researchers in diverse fields for biosensing applications. PSi is a class of photonic nanostructured materials comprising air-filled pores of diameters typically <150 nm. PSi exhibits key features for sensing and biosensing applications including fast, straightforward and cost-effective fabrication, tunable pore size and thickness, high internal surface area (up to 800m^2^ g^−1^), versatile surface chemistry, detection of biomolecules via optical reflectivity and photoluminescence, biocompatibility and biodegradability for biological applications, miniaturization, and integration in microelectromechanical systems (MEMS) and portable devices (Fischer et al., 2015). This review focuses on the recent progress in PSi hybrid biosensors using quantum dots (QDs), metal nanoparticles (MNPs), or both QDs/MNPs as nanomaterials for sensitivity enhancement and dual biosensing applications.

## Surface Chemistry

**Figure 1:**
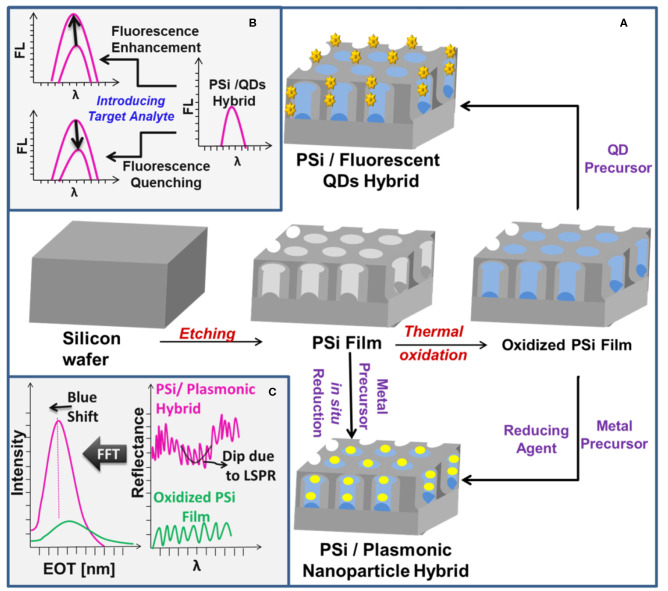
**(A)** A schematic illustration of the preparation of PSi/plasmonic MNPs and PSi/QDs hybrids. **(B,C)** Depict the optical sensing features of the prepared PSi/QDs and PSi/plasmonic MNPs hybrids, respectively.

PSi is usually fabricated by an electrochemical etching technique with subsequent thermal oxidation step which results in passivated surface with enhanced chemical stability, Figure 1A. The oxidized surface of PSi represents a key feature for developing PSi-based biosensors due to the availability of diverse grafting strategies with reactive functional groups (−NH_2_, COOH, SH, CHO) for subsequent conjugation to target biomolecules (e.g., enzymes, proteins, DNA, peptides, aptamers, drugs) (Terracciano et al., 2019). Examples of surface modification strategies are provided in the Supplementary Information document.

## Optical Biosensing Principle and Transduction Mechanisms

Optical biosensing principle and the explanation of the transduction mechanisms for various one-dimensional PSi nanostructures (single layer interferometer, Distributed Bragg Reflector (DBR), PSi microcavity (PSiMC), and Rugate filters) are provided in the Supplementary Information document with illustrations, Figures S1, S2.

### PSi-Based Hybrid Biosensors

PSi nanostructures have been used as a host matrix for preparing hybrid platforms using different nanomaterials such as QDs, MNPs, graphene oxide, carbon nanotubes, fluorescent molecules, and polymers (Arshavsky-Graham et al., 2018; Tieu et al., 2019). PSi-based hybrid platforms improve the sensitivity and stability of the sensor and allow for dual-mode detection (Gaur et al., 2013; Pacholski et al., 2019). PSi biosensors designed with single-mode optical sensing suffer from poor sensitivity limited to the micromolar range and lack of simultaneous selective detection of more than one target analyte (Massad-Ivanir et al., 2018b). In contrast, optical biosensors designed for dual-mode detection are characterized by their ability to combine the quantification and identification of molecules in a single substrate (Jiao et al., 2010; Yeom et al., 2011). The molecular quantification can be achieved *via* reflectance measurements of PSi matrix, whereas the molecular identification and conformation can be attained through Surface Enhanced Raman Spectroscopy (SERS) (Jiao et al., 2010; Pacholski et al., 2019) or LSPR (Pacholski et al., 2019) in case of using MNPs, or through fluorescence spectroscopy when fluorescent QDs are confined inside the PSi matrix (Massad-Ivanir et al., 2018a,b). The dual-mode biosensors with fluorescence or SERS characteristic enable the detection of multiple molecules with enhanced sensitivity and specificity (McNay et al., 2011). As an illustration, fluorescence enhancement/quenching signals can be achieved by careful design of the sensing environment and selection of the target analyte, Figure 1B. Besides that, incorporating QDs, which are characterized by high refractive index, has been realized as a tool for signal amplification via labeling QDs with different biomolecules and using the reflectance spectra to detect the amplified refractive index signal resulting from their interactions with the hosting PSi matrix (Lv et al., 2017; Massad-Ivanir et al., 2018b; Zhou et al., 2019). In a different scenario, incorporation of AuNPs into the PSi matrix enhances the contrast of the Fabry-P=erot fringes due to augmented surface reflectivity and consequently increases the sensitivity for both bulk refractive index sensing and unspecific/affinity biosensing, Figure 1C (Mariani et al., 2019). Interestingly, incorporating AuNPs into PSiMC has been found to enhance the fluorescence signal of the labeled probe DNA molecules upon hybridization with target DNA molecules due to the LSPR of AuNPs inside the PSiMC (Wang and Jia, 2018), Tables S1.

### PSi/Plasmonic MNPs Hybrids

Among all MNPs, AuNPs and AgNPs exhibit the most interesting physical properties for biosensing applications (Loiseau et al., 2019). AgNPs produce stronger plasmon resonance and provide higher sensitivity than AuNPs due to the large reflectivity from the metal surface (Solati and Dorranian, 2015). Regardless, AuNPs remain the most studied choice for biosensing applications due to their biocompatibility and chemical stability as compared to AgNPs which exhibit low oxidative stability in the air as well as low resistance to corrosive species present either in solution or analyte (e.g. Cl^−^) (Lin et al., 2004; McNay et al., 2011). Another attractive feature of AuNPs for biosensing applications is their high affinity to chemisorb soft binding groups such as thiol (-SH) which allow for direct conjugation and immobilization of thiol-modified bio-macromolecules (e.g. DNA) via a robust self-assembled monolayer technique, and hence, avoids the complicated multi-step surface modification procedures (McNay et al., 2011; Zhang et al., 2015, 2017).

PSi/Plasmonic AgNPs hybrids have been applied as a sensing substrate for SERS with Raman enhancement of more than ten orders of magnitude, approaching a single molecule detection (Virga et al., 2013). A recent review by Bandarenka et al. discussed the fabrication of PSi/metal nanoparticles hybrids and their application as SERS-active substrates, emphasizing two major advantages: (1) the long storage stability; and (2) the significant Raman enhancement factor (Bandarenka et al., 2018). PSi/Plasmonic nanoparticle hybrids allow for fabricating biosensors with SERS and refractive index sensing capability (Pacholski et al., 2019). Škrabi'c et al. reported the application of silver-coated PSi photonic crystals as SERS substrates for near-infrared (1064 nm) excitation, which has significant importance for the Raman detection of fragile biomolecules (Škrabić et al., 2019). Due to the non-resonant matching of the excitation wavelength with LSPR of the AuNPs deposited on the PSi, the enhanced Raman scattering was attributed to the existence of hot-spots on the sample surface. The hybrid sensor provided 5 orders of magnitude lower detection limits as compared to bare PSi, Table S1.

Wang et al. employed Rhodamine Red (RRA) fluorescent dye for labeled DNA detection using a PSiMC/AuNPs hybrid matrix with a LOD value of 10 pM (Wang and Jia, 2018), Table S1. Recently, Mariani et al. reported the fabrication of PSi/AuNPs hybrid photonic/plasmonic optical biosensor by employing a LbL self-assembly approach (Mariani et al., 2019). Compared to the traditional *in situ* reduction procedure, the self-assembling of previously prepared AuNPs onto the target surface provides a practical way to control the AuNPs' properties such as shape, dimension, and related optical/electrical features. The effect of the AuNPs' size was studied by SEM analysis and it was revealed that the smaller AuNPs with a size of 4 nm has decorated the DBR PSi scaffold with higher density throughout the whole thickness as compared to larger AuNPs with a size of 15 nm. Hence, the larger AuNPs were used to decorate the top surface of the monolayer Fabry-P =erot thin film, whereas the smaller AuNPs were employed for building the hybrid photonic/plasmonic PSi/AuNPs biosensor and decorating the inner surface of the DBR multilayer PSi nanostructure. Decoration of the monolayer Fabry-P=erot interferometer with AuNPs enhanced the contrast of the Fabry-P=erot fringes due to the increased surface reflectivity and the hybrid sensor exhibited an interferometric augmented sensitivity for bulk refractive index (≈ 4.5 folds for the infiltrated NaCl aqueous solution) and surface refractive index (≈ 2.5 folds for the unspecific adsorption of BSA and affinity biosensing of streptavidin), Table S1. In the case of hybrid photnic/plasmonic PSi/AuNPs sensor, both photonic (DBR stop-band) and plasmonic (LSPR) were shown to be sensitive to changes of bulk refractive index, with lower detection limit from the plasmonic signal, Table S1.

### PSi-Based Fluorescent Hybrids

The intrinsic photoluminescence of PSi has been used for developing sensitive biosensor through monitoring the quenching or enhancement of the PL intensity in the presence of target biomolecules such as enzymes, DNA and antibodies (Jenie et al., 2016). However, the PL measurements are susceptible to errors and interferences as the PL intensity is sensitive to the surrounding chemical environment (Tieu et al., 2019). Despite this fact, signal enhancement has been achieved by incorporating fluorescent probes such QDs, Carbon QDs (CQDs), and fluorescent molecules within various PSi nanostructures including Bragg reflectors, PSiMC and rugate filters (Arshavsky-Graham et al., 2018). In the case of PSiMC, the presence of the middle spacer layer enhances the absorption and trapping of light and allows for obtaining emission spectrum with amplified luminescence signal (Palestino et al., 2008), Figure 2B. During the past decade, QDs have been used extensively as bioaffinity probes in fluorescence-based biosensing due to their strongly diminished photobleaching, size-tunable emission wavelength, large extinction coefficients, high emission quantum yields, and long fluorescence lifetimes (Smith et al., 2008). Biosensing with QDs can be achieved by (1) making use of fluorescence resonance energy transfer (FRET); (2) Bioluminescence resonance energy transfer (BRET); (3) fluorescence quenching; and (4) changes in the fluorescence anisotropy (Smith et al., 2008; Weihs and Dacres, 2019).

**Figure 2:**
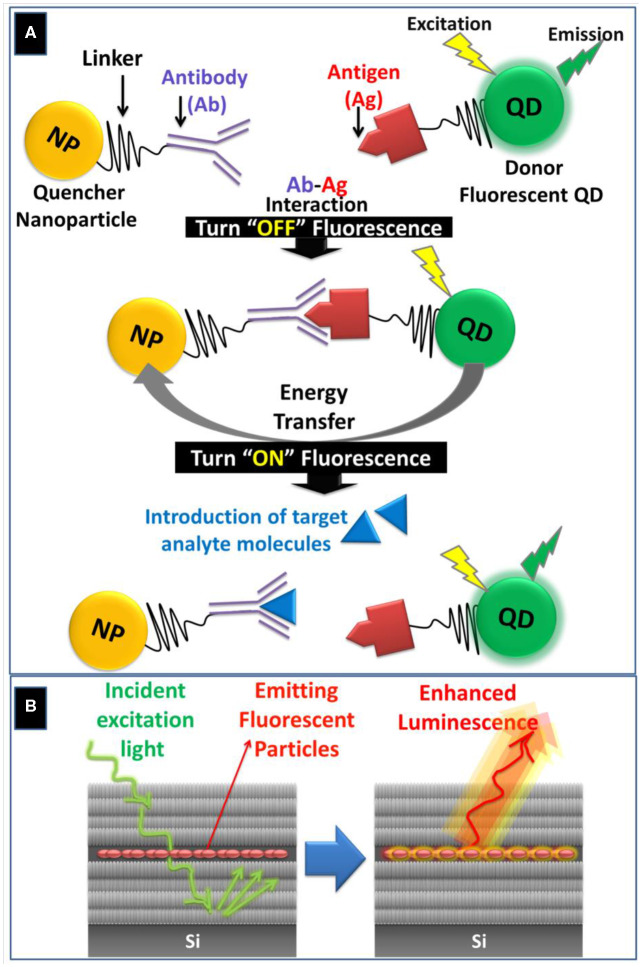
**(A)** Schematic illustration of FRET-based biosensing through quenching “Turn OFF” or activation “Turn ON” pathways using QDs-conjugated antigen and NP-conjugated antibody as an illustrative example. During the “Turn ON” stage, the target analyte molecule displaces the antigen due to its higher binding affinity with the antibody. **(B)** Schematic representation of light entrapment inside the spacer layer of PSi microcavity (PSiMC) resulting in luminescent enhancement from the embedded fluorescent particles.

Guar et al. reported a highly-sensitive dual-mode labeled detection of biotin in PSi films using QDs as signal amplifiers (Gaur et al., 2013). QDs served as (1) a high refractive index signal amplifier for the reflectance measurements for quantifying the number of molecules captured, and (2) as fluorescent emitter which allowed identification and conformation of the molecules captured (Gaur et al., 2013). Thus, QDs labeling of the target biotin molecules enabled the detection based on the distinct fluorescent signal with simultaneous enhancement in the measured spectral reflectance fringe shift (> 5-folds) and ≈ 3 orders of magnitude improvement of the detection limit for only 6% surface area coverage of QDs inside the PSi matrix (Gaur et al., 2013). Using the fluorescence detection mode, the hybrid PSi biosensor provided an exceptional biotin detection limit below 1 fg mm^−2^ and a dynamic working range of 7 pg mm^−2^ to 0.5 fg mm^−2^ (Gaur et al., 2013).

Lv et al. demonstrated the use of CdSe/ZnS QDs for improving the detection sensitivity in a PSiMC structure by amplifying the refractive index signal of the target DNA (Lv et al., 2017). The detection principle is based on the hybridization that occurs between the complementary DNA attached to PSiMC and the DNA target molecules conjugated to water-soluble QDs. The fabricated biosensor exhibited a 6-fold increase in sensitivity as compared to the detection of unlabeled DNA target. Zhou et al. reported a novel biosensing matrix based on hybrid PSiMC and fluorescent CdSe/ZnS QDs-labeled complementary DNA molecules (Zhou et al., 2019). The hybridization reaction between the PSi-grafted DNA probe and QDs-labeled complementary DNA was detected by the angular spectrum detection method. The detection limits of the QDs-labeled and label-free PSiMCs were found to be 36 pM and 10.8 nM, respectively. The higher sensitivity and lower detection limit of the QD-labeled hybrid system were attributed to the amplified refractive index signal due to the presence of fluorescent QDs conjugated to the complementary DNA molecules.

Compared to conventional semiconductors QDs, CQDs offer advantages for biosensing applications such as excellent water-solubility, good biocompatibility, low toxicity, simple synthetic routes, and tunable fluorescence emission (Loo et al., 2016). Massad-Ivanir et al. reported *in situ* synthesis of CQDs in a PSi Bragg reflector matrix with fine-tuned and modulated optical properties by controlling the nanostructure architecture of the hosting PSi matrix (Massad-Ivanir et al., 2018a). It was observed that the fluorescence signal of CQDs can be enhanced significantly when the high reflection band of the PSi host overlaps with the confined C-dots' peak wavelength, and the PSi matrix is thermally oxidized at mild conditions (Massad-Ivanir et al., 2018a).

In a different study, Massad-Ivanir et al. reported a novel fabrication of a bimodal sensing platform comprising CQDs integrated within a PSi host matrix through one step *in situ* synthetic pathway (Massad-Ivanir et al., 2018b). The hybrid PSi/CQDs allowed for dual-mode detection using the reflectivity of the nanostructured PSi and the fluorescence of the embedded CQDs, with superior sensing performance compared to that of individual components. The resulted hybrid nanostructured Fabry–Pérot PSi thin film was employed for label-free optical detection of trypsin and adenosine triphosphate (ATP). Compared to the results obtained by using the reflectance mode, lower detection limits were attained with a linear response when the fluorescence mode was used. The incorporation of CQDs allowed for real-time detection of small molecules such as ATP with concentrations as low as 0.1 mM, which presents a detection limit with one order of magnitude lower than those of neat PSi film or free C-dots solution. The enhanced sensitivity of CQDs was attributed to the nanoporous environment of the hosting PSi matrix which promotes the interactions between the carbon nanoparticles and the adsorbed analytes. Simultaneous dual-mode detection of trypsin (43 μm) was demonstrated using a customized setup for simultaneous fluorescence imaging and optical reflectivity spectra recording.

### PSi-Based Plasmonic and Fluorescent Hybrids

Fluorescence resonance energy transfer (FRET) is a non-radiative energy transfer from an excited energy donor to an acceptor via long-range dipole-dipole interactions (Hou et al., 2020). FRET-induced fluorescence quenching occurs when fluorescence emission band of the donor fluorophores (e.g., QDs) overlaps with the absorption band of the quencher nanoparticles (e.g., AuNPs) (Wu et al., 2017). The former FRET-based biosensing depends on the inhibition or “turn off” of the fluorescence signal, Figure 2A. Alternatively, the “turn-on” assay is another FRET-based strategy for detecting biomolecules by adding a target analyte that displaces the quencher, which leads to a proportional recovery of the fluorescence signal (Anfossi et al., 2018), Figure 2A.

Recently, FRET has been exploited as an alternative approach for sensitivity enhancement using fluorescent QDs and AuNPs through DNA hybridization within a PSi Brag reflector host (Zhang et al., 2017). The biosensor was tested for detection of 16S rRNA based on quenching of the PL emission of QDs-conjugated onto the PSi layer by AuNPs-conjugated complementary 16S rRNA. The fabricated biosensor exhibited a linear response for the target DNA in the concentration range from 0.25 to 10 μM with a LOD value of ≈ 328.7 nM. Zhang et al. reported a new type of fluorescent “off-on” probe for *L*-cysteine based on FRET between amino-capped porous silicon nanoparticles (PSiNPs) and citrate-stabilized AuNPs with a LOD value of 35 μm (Zhang and Jia, 2017).

## Conclusions and Future Perspectives

The continuous research during the past two decades led to establishing the basic technology of PSi-based biosensors with stable chemical structure, versatile surface modification, and applications in areas such as medical diagnostics, food safety, homeland security, and environmental monitoring. Recently, more research focus has been devoted to evaluating new PSi-based hybrid platforms for sensitivity enhancement, signal amplification, and generation of dual-mode biosensors. Among various nanomaterial, MNPs and QDs are promising candidates for preparing hybrid ultrasensitive PSi-based biosensors, Table S1. Incorporation of MNPs allows for dual-mode detection such as in the case of SERS, whereas the use of QDs enables the development of biosensors with dual reflectance and fluorescence signals, Table S1. MNPs and QDs can be used together for developing FRET-based assay biosensors by monitoring the inhibition or reactivation of fluorescence signal upon the introduction of target biomolecules, Table S1. The use of hybrid PSi-based biosensors allowed for shifting the limits of detections (LODs) from micromolar range to nanomolar range for unlabeled targets, whereas LODs in the pico- and femtomolar ranges were achieved in case of labeled target analytes, Table S1. Future studies shall focus on real sample analysis and integrated biosensor devices.

## Author Contributions

All authors listed have made a substantial, direct and intellectual contribution to the work, and approved it for publication.

## Conflict of Interest

The authors declare that the research was conducted in the absence of any commercial or financial relationships that could be construed as a potential conflict of interest.
