# Supplementary material for: Hybrid Porous Silicon Biosensors Using Plasmonic and Fluorescent Nanomaterials: A Mini Review
Source: Front Chem. 2020 May 29;8:454. doi: 10.3389/fchem.2020.00454 (PMC7272471; doi:10.3389/fchem.2020.00454)
Supplement: Supplementary file 1 [file Data_Sheet_1.pdf]

Supplementary Information

Hybrid Porous Silicon Biosensors Using Plasmonic and Fluorescent Nanomaterials: A Mini Review

Nedal Abu-Thabit[1] and Elaref Ratemi[1]*

[1]*Department of Chemical and Process Engineering Technology, Jubail Industrial College, Jubail Industrial City, Al Jubail, Saudi Arabia*.

***Author to whom correspondence should be addressed; E-Mail: abuthabit_nidal@yahoo.com**

1. Surface Chemistry of PSi:

PSi is usually fabricated by an electrochemical etching technique with subsequent thermal oxidation step which results in passivated surface with enhanced chemical stability, **Figure 1 (A)**. The oxidized surface of PSi with (Si-OH) groups is considered as a key features for developing PSi-based biosensors due to the availability of diverse strategies for grafting the surface of PSi with various reactive functional groups (-NH2, COOH, SH, CHO) for subsequent conjugation of biomolecules (e.g. enzymes, proteins, DNA, peptides, aptamer, drugs) (Terracciano et al., 2019).

Hydrolytic condensation has been employed successfully for grafting the surface of PSi with silane coupling agents such as 3-aminopropyltriethoxy-silane (APTES) and 3-mercaptopropyltriethoxy-silane (MPTES) which allow for the conjugation of biomolecules through amide and thiol-ene coupling reactions, respectively (Lee et al., 2018). Another attractive feature of using APTES is its ability to form ionic interactions with stable ionic bond through the electron pair of (-NH_2), which facilitates the immobilization of functional nanomaterials such as quantum dots into the surface of PSi matrix (Gaur et al., 2013).

The original reduced from PSi surface with (Si-H) groups have been used for bioconjugation through a different pathways such as grafting PSi surface with COOH-terminated alkenes *via* thermal/ UV hydrosilylation, followed by activation with EDC/NHS, and finally immobilization of biomolecules by coupling with the formed succinimidyl ester group (Reta et al., 2016, Rossi et al., 2007). Another interesting feature of the PSi surface with (Si-H) groups is its ability for *in situ* reduction of metal ions and formation of MNPs such as gold (AuNPs) and silver (AgNPs) nanoparticles (Lin et al., 2004), **Figure 1 A**.

A novel approach for functionalization of PSi surface employed electrostatic Layer-by-Layer (LbL) nano-assembly of oppositely-charged polyelectrolytes, engineered with bioreceptors for label-free detection of target analytes (Mariani et al., 2018). Recently, the versatility of the LbL technique has been utilized for fabricating hybrid PSi-based biosensor with photonic/ plasmonic dual-mode detection *via* incorporation of negatively charged citrate-capped AuNPs in the deposited self-assembled polyelectrolyte multilayer (PEM) nanofilm (Mariani et al., 2019).

2. Biosensors based on Nanostructured PSi :

A biosensor is a device that consists of at least two parts: (1) a molecular recognition element (receptor) that selectively interacts with a specific target analyte (e.g. DNA, antibodies, enzymes, cells microorganisms); and (2) a physicochemical transducer that converts the bio-recognition information into a measurable electrochemical, electrical, magnetic, thermal or optical signal. Biosensors can be designed to operate with label or label-free detection modes. In the former case, the target analytes are labeled with fluorescent, enzymatic or radioactive tags. Although this method provides high sensitivity and allows for even a single-molecule detection, the development of label-free detection techniques has attracted researches for designing simple, fast and cost-effective biosensors for various applications (Soler et al., 2019, Chocarro-Ruiz et al., 2017, Khansili et al., 2018). The distinctive and tunable photonic properties of PSi allow for the design and fabrication of label-free biosensors for diverse applications (Reta et al., 2019, Terracciano et al., 2019). The sensing principle of PSi nanostructures is based on monitoring the modification of transducer response (optical, electrical, thermal, chemical or electrochemical) through “surface-target biomolecules” interaction

in real-time or express detection (Myndrul and Iatsunskyi, 2019). This review focuses only on the PSi-based optical biosensors and their transduction mechanism is discussed.

2.1 Optical Transduction:

PSi-based optical biosensors can be classified into three main groups based on the transduction mechanism: (1) Luminescent biosensors based on monitoring the changes in photoluminescence/ fluorescence signal; (2) Reflectance biosensors based on monitoring the shift in the fringe pattern (Δλ) resulting from changes in the average refractive index of the PSi matrix; and (3) colorimetric optical biosensors based on visual observation by naked eye.

White light is shone into the PSi structure, which is reflected on the two interfaces (Air/PSi and PSi-bulk Si) of the porous silicon structure, producing a Fabry-Pērot interference fringe pattern with interference maxima located at the wavelength (λ_m), as per the following equation:

$$m\,\lambda_m = \frac{2n_{ef}L}{Cos\ \theta} \qquad (1)$$

Where *m* is the spectral order of a fringe, *θ* is the angle of the incident light, n_{ef} is the refractive index of the effective medium of the porous layer, and *L* is the physical thickness in nm. Thus, the position of an interference maximum λ_m is proportional to the refractive index of the surrounding medium n_{ef}, **Figure S1 (A)**. The term ($2n_{ef}$ L) is so-called effective optical thickness (EOT), which can be tuned by adjusting the porosity and thickness of PSi film. The EOT is usually used as to express the sensor response, and can be calculated from the slope of the straight line resulted from the plot of *m* vs. (1/ λ_m) (Pacholski et al., 2019). Another convenient way to determine the EOT is by

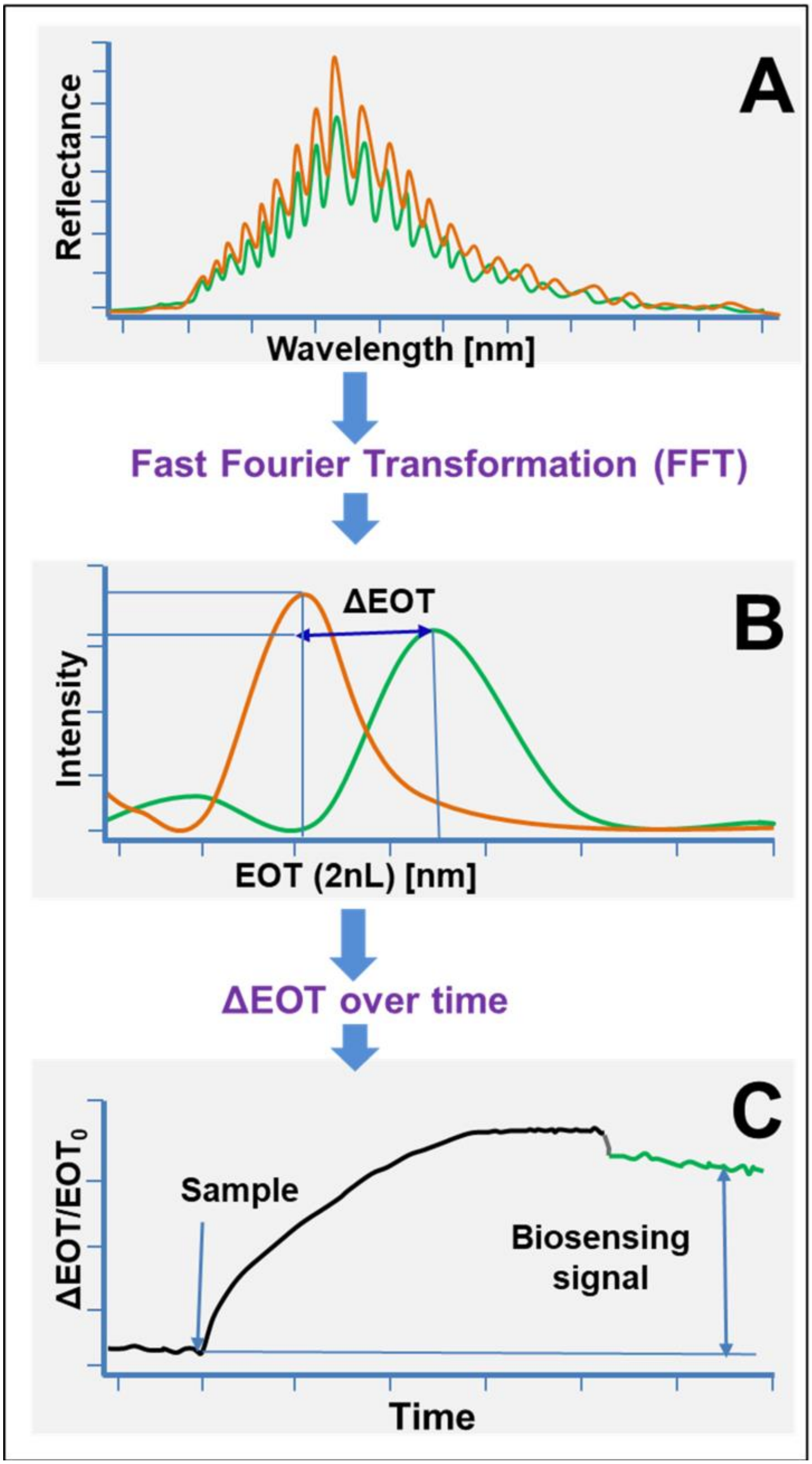

Figure S1 (**A-C)** A schematic illustration of the optical sensing principle of PSi using the reflectance spectroscopy

applying Fast Fourier Transform (FFT) to the reflectance spectrum in wave numbers. The resulting graph of the intensity vs. EOT displays a peak at the position of the highest intensity corresponding to the magnitude of EOT in nm (Pacholski et al., 2019), **Figure S1 (B)**. Analytes are detected and quantified based on continuous monitoring of the change in EOT signal with time, **Figure S1 (C)**.

3. Types of PSi structures

3.1 Single layer interferometer

The simplest and the most reported geometry of PSi is a single layer interferometer where a constant current density is applied during the electrochemical etching process. As shown in **Figure S2 (A),** Fabry-Pērot fringes are obtained from the interference of the white light reflecting off the top and bottom interfaces of PSi. A constructive interference occurs when the conditions in equation 1 are fulfilled.

3.2 Bragg Reflectors:

PSi can be fabricated with a bi-layer structure by changing between low and high current densities during the etching process. When the process is repeated many times, it produces PSi with multilayer structures known as distributed Bragg reflectors (DBR) or Bragg mirrors, comprised of periodically stacked high refractive index layer (layer H) and low refractive index layer (layer L), each having an EOT of λ/4 in the middle of the high reflectance stop band as illustrated in **Figure S2 (B)** and described in equation 2:

$$\frac{\lambda_{\circ}}{4n} = L_{mirror} \qquad (2)$$

The reflectance spectrum of the Bragg mirror structures is featured with higher harmonics, called side lobes, that are located at integer multiples of the energy of the first stop band, **Figure S2 (B).**

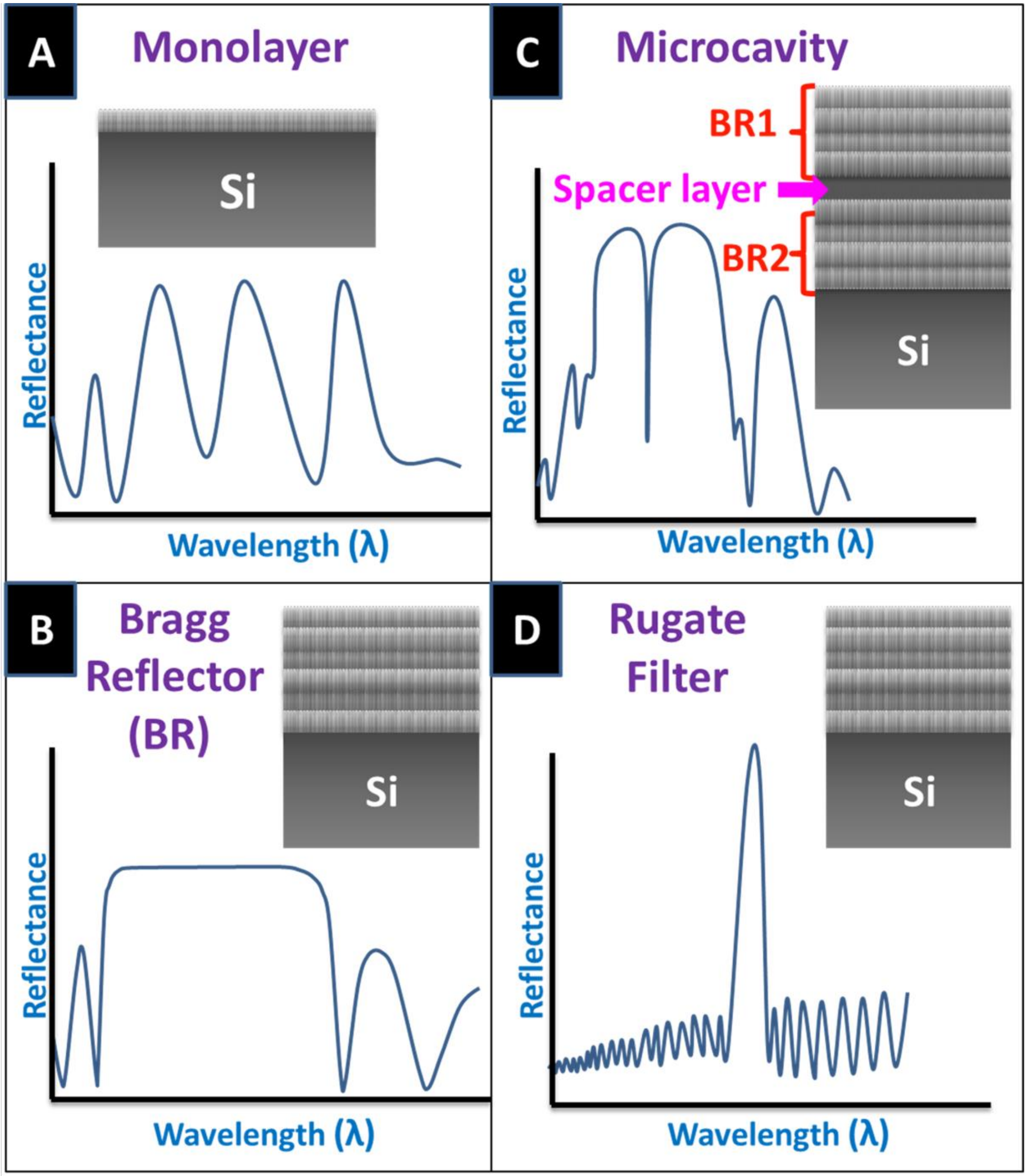

Figure S2 (A-D) Common one dimensional PSi structures and their associated optical spectra

3.3 Microcavity:

During the etching process, PSi microcavity (PSiMC) is generated by first forming the top Bragg mirror made from alternating H and L layers. This step is followed by changing the anodisation conditions to form a middle spacer layer and at the end formation of the bottom Bragg mirror, **Figure S2 (C)**. The quality of the energy stored within the PSiMC structure can be determined according to the following equation:

$$Q = \frac{\lambda}{\Delta\lambda} \qquad (3)$$

Where Q is the quality factor, λ is the resonance wavelength and $\Delta\lambda$ is defined as the full width half maximum (FWHM) of the transmittance peak. As the value of quality factor increases, the energy loss from PSiMC becomes smaller. PSiMCs with high Q values can be obtained by increasing the porosity or refractive index contrast between the alternating H and L layers. The reflectance spectrum of the PSiMC is characterized by a narrow dip with FWHM less than 10 nm, and large reflectance stop bands. The position of the resonance wavelength can be adjusted by tuning the thickness of the H and L layers, **Figure S2 (C)**.

3.4 Rugate Filters:

Rugate filters are structures in which the refractive index varies smoothly and periodically in depth. Rugate filters are produced by applying sinusoidal current density waveform for number of repeated cycles with certain periodicity. Hence, the obtained PSi structure possesses an approximately sinusoidal porosity which results in a periodic variation in the refractive index of the PSi film. The continuous variation of the refractive index profiles produces a sharp diffraction feature in the optical reflectivity spectrum and helps to suppress the side lobes that are present in the reflectance spectra of the

microcavity and Bragg mirror (Arshavsky-Graham et al., 2018)**, Figure S2 (D)**. Rugate filters can be designed with small Δn and many periods to achieve a narrow-width and high contrast reflectance peak according to the following equation (Arshavsky-Graham et al., 2018):

$$\mathrm{n(x)} = n_{\circ} + \frac{\Delta n}{2} \sin\left(\frac{4nx}{\lambda_{\circ}}\right) \qquad (4)$$

Table S1 Performance summary of biomolecules detection using different hybrid PSi-based biosensors

Type	Hybrid PSi Structure	Fabrication method	Target Analyte	Transduction Mechanism	Label-Free/ Labeled	Detection Range/ Tested Concentrations	LOD/ Sensitivity	Ref.
PSi/ Metal Nanoparticles (MNPs)	Ag-coated PSi Photonic Crystal	*in situ* reduction of $AgNO_3$	Rhodamine 6G (R6G) dye	SERS	Label-free	0.1 nM to 10^6 nM	0.1 nM	**(Zhong et al., 2018)**
	Ag-coated PSi Photonic Crystal	*in situ* reduction of $AgNO_3$	Picric Acid	SERS	Label-free	100 nM to 10^5 nM	10 nM	**(Škrabić et al., 2019)**
			Rhodamine 6G (R6G) dye	SERS	Label-free	10 nM to 10^6 nM	100 nM	
	Ag-coated PSi disks	*in situ* reduction of $AgNO_3$	crystal violet (CV) dye	SERS	Label-free	10 nM to 10^6 nM	50 nM	**(Bu et al., 2017)**
			Glutathione	SERS	Label-free	< 568,9 nM	74.9 nM	
	PSiMC/ AuNPs/ Rhodamine red (RRA)	Hydrothermal synthesis using $HAuCl_4$. Then immobilized into H_2N-PSi. [a]	DNA	Reflectivity / Fluorescence	Labeled probe[a]	10 μM to 10^{-4} μM	10 pM	**(Wang and Jia, 2018)**
	PSiMC/Au	*in situ* reduction of $HAuCl_4$	ssDNA	Fluorescence	Labeled Target	10^{-4} μm to 10 μm	10 pM	**(Wang and Jia, 2018)**
	PSiMC/Au	*in situ* reduction of $HAuCl_4$	ssDNA	Reflectivity	Label-free	2-10 μm	15.15 nM	**(Zhang et al., 2015)**
	Fabry-Pérot thin film/Au nanocomposite	*in situ* reduction of $HAuCl_4$ and Electrochemical reduction	Aflatoxin B1	PL	Label-free	0.01 ng/mL to 5 ng/mL	2.5 pg/mL	**(Myndrul et al., 2017)**
	Distributed Brag Reflector / Au nanoparticle size = 4 nm)	*LbL self- assembly*	Glucose	Reflectivity	Label-free	35% Glucose solution	Plasmonic signal is 27 folds greater than photonic signal	**(Mariani et al., 2019)**
			BSA	Reflectivity	Label-free	20 – 2000 μg/mL	Plasmonic (20 μg/mL) Photonic (200 μg/mL)	
	PSi (Fabry-Pérot thin film) /Au (nanoparticle size = 15 nm)	*LbL self- assembly*	BSA	Reflectivity + IAW signal processing	Label-free	1000 μg/mL	5.39 au (2.04 au, for PSi without Au)	**(Mariani et al., 2019)**
			NaCl	Reflectivity + IAW signal processing	Label-free	1 – 10 % w/v	6.2 x10^{-6} RIU (2.85 x10^{-5} RIU, for PSi without Au)	
			Streptavidin	Reflectivity + IAW signal processing	Label-free	500 μg/mL	1.88 au (0.77au, for PSi without Au)	

PSi/ Quantum Dots (QDs)	PSi (Fabry-Pérot thin film)/C-dot	*in situ formation of C-dots by thermal reduction of glucose solution.*	Trypsin	Reflectivity & Fluorescence	Label-free	4.3 μM to 43 μM	4 μm	**(Massad-Ivanir et al., 2018)**
			ATP		Label-free	0.1 mM to 10 mM	0.1 mM	
	PSi (Fabry-Pérot thin film)/ coupled to QDs	Immobilization of QD-conjugated biotin into PSi	Biotin	Reflectivity & Fluorescence	Labeled[b]	7 pg mm^{-2} to 0.5 fg mm^{-2}	1 fg mm^{-2}	**(Gaur et al., 2013)**
	PSiMC/QDs	Immobilization of the complementary DNA into PSi Matrix.	DNA	Reflectivity	Labeled[c]	0.1 μM to 5.0 μM	6.97 nM	**(Lv et al., 2017)**
	PSiMC/QDs	Immobilization of the target DNA into PSi matrix.	DNA	Angular spectrum detection method	Labeled[d]	0.05 nM – 1 nM	36 pM	**(Zhou et al., 2019)**
PSi/ AuNPs/ Fluorescent Particles	PSi Brag reflector/QDs/AuNPs	Immobilization of the QDs-conjugated probe DNA into PSi matrix.	16S rRNA	PL	Label-free[e]	0.25 μM to 10 μM	328.7 nM	**(Zhang et al., 2017)**
	Fluorescent H_2N-capped PSi nanoparticles/ AuNPs.	See below[e]	L-cysteine	PL	Label-free[f]	0.125 mM to 5 mM	35 μm	**(Zhang and Jia, 2017)**

[a] Firstly, the PSiMC were functionalized to adsorb AuNPs; secondly, the thiol modified DNA was connected with the AuNPs as a target DNA; then, complementary DNA modified with RRA (probe DNA) was hybridized with target DNA. The detection is based on fluorescence enhancement triggered by hybridization between the target DNA and the RRA-labeled probe DNA.

[b] QDs are considered as labeling agent for biotin target molecule; streptavidin was used as probe molecules and it was immobilized on the PSi matrix.

[c] QDs are considered as labeling agent for the target DNA molecules; complementary DNA molecules were conjugated to PSi surface as probe molecules. The detection is based on hybridization between the target DNA and the complementary DNA.

[d] QDs are considered as labeling agent for the complementary DNA molecules; target DNA molecules were conjugated to PSi surface as probe molecules. The detection is based on hybridization between the target DNA and the complementary DNA.

[e] QDs act as an emission donor and AuNPs serve as a fluorescence quencher. QDs were conjugated to probe DNA, and citrate capped AuNPs were conjugated to target DNA. The detection is based on PL quenching triggered by hybridization between the target DNA and the probe DNA.

[f] Water soluble amino-conjugated PSi nanoparticles in ethanol with excellent PL properties act as the energy donor; citrate capped AuNPs act as fluorescence quencher (Turn off emission). The restoration of emission signals (Turn on) is triggered by addition of L-cysteine target analyte. The concentration of L-cysteine is proportional to the restored PL signal.

4. References:

ARSHAVSKY-GRAHAM, S., MASSAD-IVANIR, N., SEGAL, E. & WEISS, S. 2018. Porous silicon-based photonic biosensors: Current status and emerging applications. *Analytical chemistry,* 91**,** 441-.467

D., LIU, X. & HU, Z. 2017. Silver- ,BU, Y., ZHU, G., LI, S., QI, R., BHAVE, G., ZHANG, D., HAN, R., SUN Nanoparticle-Embedded Porous Silicon Disks Enabled SERS Signal Amplification for Selective .Glutathione Detection. *ACS applied nano materials,* 1**,** 410-417

M. 2017. Nanophotonic .CHOCARRO-RUIZ, B., FERNÁNDEZ-GAVELA, A., HERRANZ, S. & LECHUGA, L label-free biosensors for environmental monitoring. *Current opinion in biotechnology,* 45**,** 175-.183

GAUR, G., KOKTYSH, D. S. & WEISS, S. M. 2013. Immobilization of Quantum Dots in Nanostructured Porous Silicon Films: Characterizations and Signal Amplification for Dual-Mode Optical .Biosensing. *Advanced Functional Materials,* 23**,** 3604-3614

KHANSILI, N., RATTU, G. & KRISHNA, P. M. 2018. Label-free optical biosensors for food and biological .*Chemical,* 265**,** 35-49 :sensor applications. *Sensors and Actuators B*

LEE, S. H., KANG, J. S. & KIM, D. 2018. A mini review: Recent advances in surface modification of porous .silicon. *Materials,* 11**,** 2557

LIN, H., MOCK, J., SMITH, D., GAO, T. & SAILOR, M. J. 2004. Surface-enhanced Raman scattering from .silver-plated porous silicon. *The Journal of Physical Chemistry B,* 108**,** 11654-11659

LV, C., JIA, Z., LV, J., ZHANG, H. & LI, Y. 2017. High Sensitivity Detection of CdSe/ZnS Quantum Dot-.80 ,7Labeled DNA Based on N-type Porous Silicon Microcavities. *Sensors,* 1

MARIANI, S., PAGHI, A., LA MATTINA, A. A., DEBRASSI, A., DÄHNE, L. & BARILLARO, G. 2019. Decoration of Porous Silicon with Gold Nanoparticles via Layer-by-Layer Nanoassembly for Interferometric and Hybrid Photonic/Plasmonic (Bio) sensing. *ACS applied materials & interfaces,* 11**,** 43731-.43740

MARIANI, S., ROBBIANO, V., STRAMBINI, L. M., DEBRASSI, A., EGRI, G., DÄHNE, L. & BARILLARO, G. 2018. Layer-by-layer biofunctionalization of nanostructured porous silicon for high-sensitivity and .high-selectivity label-free affinity biosensing. *Nature communications,* 9**,** 1-13

MASSAD-IVANIR, N., BHUNIA, S. K., RAZ, N., SEGAL, E. & JELINEK, R. 2018. Synthesis and characterization of a nanostructured porous silicon/carbon dot-hybrid for orthogonal molecular detection. *NPG* .*Asia Materials,* 10**,** e463-e463

MYNDRUL, V. & IATSUNSKYI, I. 2019. Nanosilicon-Based Composites for (Bio) sensing Applications: .Current Status, Advantages, and Perspectives. *Materials,* 12**,** 2880

MYNDRUL, V., VITER, R., SAVCHUK, M., KOVAL, M., STARODUB, N., SILAMIĶELIS, V., SMYNTYNA, V., RAMANAVICIUS, A. & IATSUNSKYI, I. 2017. Gold coated porous silicon nanocomposite as a substrate for photoluminescence-based immunosensor suitable for the determination of .Aflatoxin B1. *Talanta,* 175**,** 297-304

PACHOLSKI, C., BALDERAS-VALADEZ, R. F. & SCHÜRMANN, R. 2019. One Spot–Two Sensors: Porous Silicon Interferometers in Combination with Gold Nanostructures showing Localized Surface .Plasmon Resonance. *Frontiers in chemistry,* 7**,** 593

C., PRIETO-SIMÓN, B. & VOELCKER, N. H. 2016. Porous silicon ,RETA, N., MICHELMORE, A., SAINT membrane-modified electrodes for label-free voltammetric detection of MS2 bacteriophage. .*Biosensors and Bioelectronics,* 80**,** 47-53

RETA, N., MICHELMORE, A., SAINT, C. P., PRIETO-SIMON, B. & VOELCKER, N. H. 2019. Label-free bacterial toxin detection in water supplies using porous silicon nanochannel sensors. *ACS sensors,* 4**,** .1515-1523

ROSSI, A. M., WANG, L., REIPA, V. & MURPHY, T. E. 2007. Porous silicon biosensor for detection of .viruses. *Biosensors and Bioelectronics,* 23**,** 741-745

ŠKRABIĆ, M., KOSOVIĆ, M., GOTIĆ, M., MIKAC, L., IVANDA, M. & GAMULIN, O. 2019. Near-infrared surface-enhanced Raman scattering on silver-coated porous silicon photonic crystals. .*Nanomaterials,* 9**,** 421

SOLER, M., HUERTAS, C. S. & LECHUGA, L. M. 2019. Label-free plasmonic biosensors for point-of-care .diagnostics: a review. *Expert review of molecular diagnostics,* 19**,** 71-81

.TERRACCIANO, M., REA, I., BORBONE, N., MORETTA, R., OLIVIERO, G., PICCIALLI, G. & DE STEFANO, L Porous Silicon-Based Aptasensors: The Next Generation of Label-Free Devices for Health .2019 .Monitoring. *Molecules,* 24**,** 2216

WANG, J. & JIA, Z. 2018. Metal nanoparticles/porous silicon microcavity enhanced surface plasmon .resonance fluorescence for the detection of DNA. *Sensors,* 18**,** 661

ZHANG, H. & JIA, Z. 2017. Development of fluorescent FRET probes for “off-on” detection of L-cysteine based on gold nanoparticles and porous silicon nanoparticles in ethanol solution. *Sensors,* 17**,** .520

LV, X. 2015. Surface layer reflective index changes of Au nanoparticle functionalized & .ZHANG, H., JIA, Z .porous silicon microcavity for DNA detection. *Current Applied Physics,* 15**,** 870-876

between quantum dots ZHANG, H., LV, J. & JIA, Z. 2017. Efficient fluorescence resonance energy transfer and gold nanoparticles based on porous silicon photonic crystal for DNA detection. *Sensors,* 17**,** .1078

ZHONG, F., WU, Z., GUO, J. & JIA, D. 2018. Porous silicon photonic crystals coated with Ag nanoparticles .as efficient substrates for detecting trace explosives using SERS. *Nanomaterials,* 8**,** 872

ZHOU, R., JIA, Z., LV, X. & HUANG, X. 2019. The Enhanced Sensitivity of a Porous Silicon Microcavity .Biosensor Based on an Angular Spectrum Using CdSe/ZnS Quantum Dots. *Sensors,* 19**,** 4872
